# Exploring causal associations of antioxidants from supplements and diet with attention deficit/hyperactivity disorder in European populations: a Mendelian randomization analysis

**DOI:** 10.3389/fnut.2024.1415793

**Published:** 2024-09-24

**Authors:** Jing Chen, Lifei Chen, Xinguang Zhang, Wenbo Yao, Zheng Xue

**Affiliations:** ^1^Department of Pediatrics, Shanghai Municipal Hospital of Traditional Chinese Medicine, Shanghai University of Traditional Chinese Medicine, Shanghai, China; ^2^Shanghai University of Traditional Chinese Medicine, Shanghai, China; ^3^Shanghai Municipal Hospital of Traditional Chinese Medicine, Shanghai University of Traditional Chinese Medicine, Shanghai, China

**Keywords:** antioxidant, attention deficit/hyperactivity disorder, Mendelian randomization, green tea, dietary

## Abstract

**Background:**

Antioxidants from both supplements and diet have been suggested to potentially reduce oxidative stress in individuals with ADHD. However, there is a lack of studies utilizing the Mendelian randomization (MR) method to explore the relationship between dietary and supplemental antioxidants with ADHD.

**Methods:**

This study employed two-sample mendelian randomization. Various specific antioxidant dietary supplements (such as coffee, green tea, herbal tea, standard tea, and red wine intake per week), along with diet-derived circulating antioxidants including Vitamin C (ascorbate), Vitamin E (*α*-tocopherol), Vitamin E (*γ*-tocopherol), carotene, Vitamin A (retinol), zinc, and selenium (*N* = 2,603–428,860), were linked to independent single nucleotide polymorphisms (SNPs). Data on ADHD was gathered from six sources, comprising 246,888 participants. The primary analytical method utilized was inverse variance weighting (IVW), with sensitivity analysis conducted to assess the robustness of the main findings.

**Results:**

In different diagnostic periods for ADHD, we found that only green tea intake among the antioxidants was significantly associated with a reduced risk of ADHD in males (OR: 0.977, CI: 0.963–0.990, *p* < 0.001, FDR = 0.065), with no evidence of pleiotropy or heterogeneity observed in the results. Additionally, a nominal causal association was found between green tea intake and childhood ADHD (OR: 0.989, 95% CI: 0.979–0.998, *p* = 0.023, FDR = 0.843). No causal relationships were detected between the intake of other antioxidant-rich diets and ADHD.

**Conclusion:**

Our study found a significant inverse association between green tea intake and male ADHD, suggesting that higher green tea consumption may reduce ADHD risk in males. Further research is needed to explore optimal doses and underlying mechanisms.

## 1 Introduction

Attention deficit hyperactivity disorder (ADHD) is a prevalent neurodevelopmental condition characterized by symptoms of inattention, hyperactivity, and impulsivity (1). According to epidemiological data, 5.3% of children under the age of 18 worldwide have ADHD (2). Despite beginning in childhood, symptoms frequently last throughout adulthood, impairing social, intellectual, and occupational functioning (3).

Research on ADHD has mostly ignored other significant aspects, such as oxidative metabolism, in favor of heredity. The involvement of oxidative stress in ADHD has been highlighted by recent research (4, 5), wherein an excess of reactive oxygen species (ROS) damages neurons and disrupts cellular processes. Short-lived ROS can attach to proteins, lipids, and DNA, influencing the activity of enzymes and neurotransmitters (6, 7). Numerous neurological problems, including mental illnesses, are associated with neuronal death due to this oxidative damage (8, 9). The brain, characterized by its high fat content, is particularly susceptible to oxidative stress due to peroxidizable fatty acids, heightened mitochondrial activity, limited antioxidant defenses, and robust glucose uptake, all of which contribute to increased free radical generation (10–12). Antioxidants are crucial for balancing free radicals and preventing oxidative stress (13), which contributes to neurological disorders (14, 15). Studies suggest that increasing antioxidant intake through nutrition may further protect neurons by enhancing free radical scavenging (16, 17).

Antioxidants, in conjunction with the endogenous oxidase system, may be a useful strategy for treating ADHD by reducing oxidative damage, according to reports (18). The function of dietary antioxidants in the treatment of ADHD has been well investigated. When compared to their healthy counterparts, children with ADHD have significantly reduced levels of micronutrients including zinc and copper, which are essential for antioxidant defense (19). Further, circulating trace copper levels may provide protection against ADHD, according to a Mendelian randomization study (20). Additionally, the essential antioxidant enzyme Superoxide Dismutase (SOD) activity seems to be lower in ADHD patients (21). On the other hand, vitamin E, well known for its capacity to lower lipid peroxidation, has demonstrated potential in the management of ADHD (22).

Mendelian randomization (MR), a technique utilized to infer potential causality, has been widely used to assess the relationship between risk factors and disease occurrence. The foundation of MR is the random assignment of alleles from parents to children, which guarantees that the disease has no effect on the development of fertilized eggs or fixed genotypes. This strategy aids in reducing problems with reverse causality and mixed bias (23). In this study, we explored the relationship between circulating antioxidants derived from diet [Vit. C (ascorbate), Vit. E (*α*-tocopherol), Vit. E (*γ*-tocopherol), Carotene, Vit. A (retinol), Zinc, and Selenium] and dietary habits (coffee, tea, and red wine) with the risk of ADHD using a thorough MR analysis at the genetic level.

## 2 Methods

### 2.1 Study design

**Figure 1:**
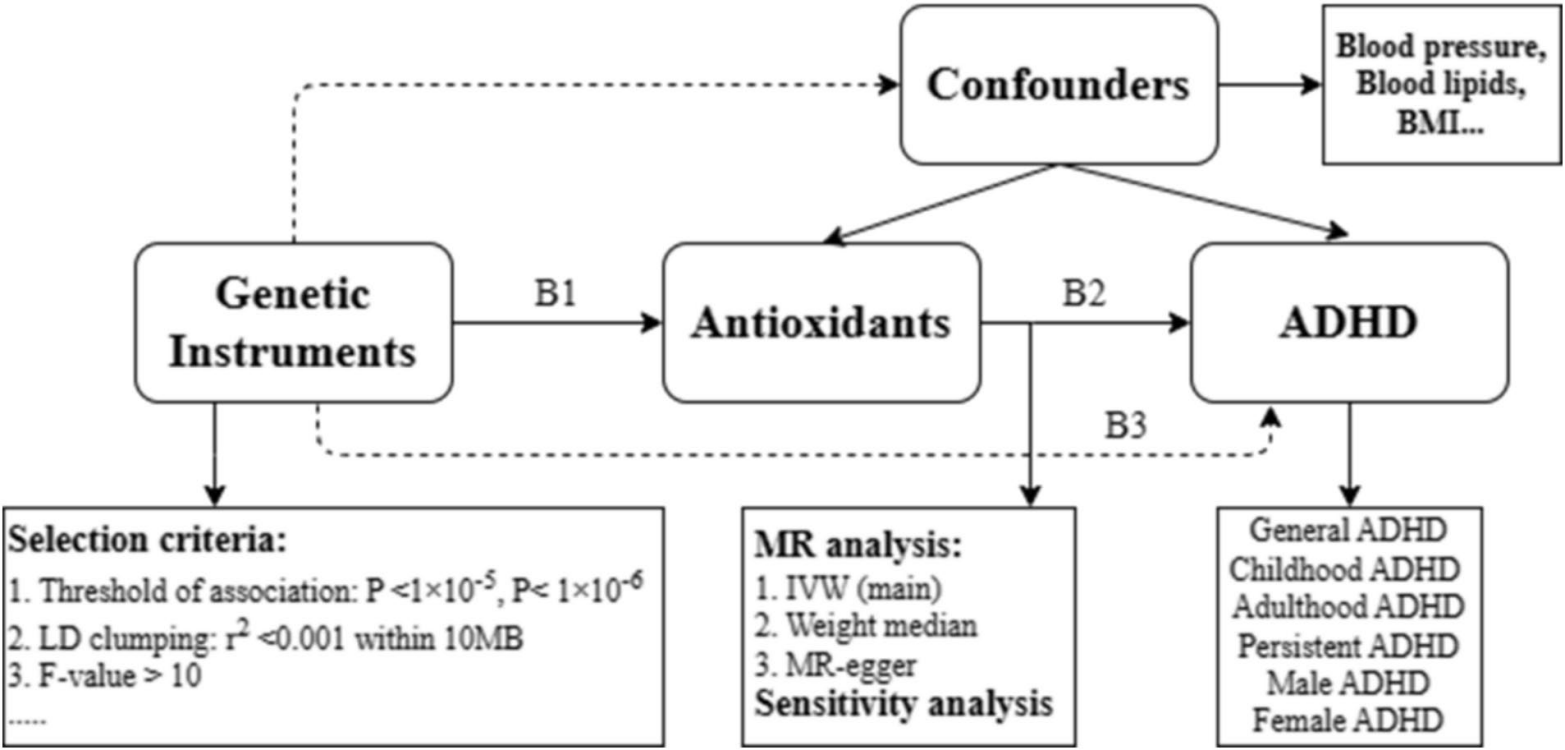
Drawing of the study design. All summary-level GWAS datasets were derived primarily from subjects of European descent. The causal association of interest (antioxidants on ADHD) is estimated using the ratio B2 = B3/B1, where B1 represents the genetic association with exposure (antioxidants), and B3 represents the genetic association with the outcome (ADHD). ADHD: attention deficit/hyperactivity disorder. IVW: inverse variance weighted; BMI: body mass index.

The current study used summary-level data to conduct an MR analysis. The use of genetic instrumental variables that simulate a randomized control trial (RCT) is the fundamental idea of MR causality analysis. A MR analysis also depends on the subsequent three presumptions (24): (1) genetic instruments (SNPs) exhibit a robust association with the exposure, (2) genetic instruments are independent of potential confounders, and (3) genetic instruments influence outcomes solely through the exposure (Figure 1).

### 2.2 Exposure of GWAS data

Antioxidant dietary supplements (coffee, green tea, herbal tea, standard tea, and average weekly red wine intake) and diet-derived circulating antioxidants [vitamin C (ascorbate), vitamin E (*γ*-tocopherol), vitamin A (retinol), carotene, zinc, and selenium] were the exposure factors taken into consideration in this study. The UK Biobank (UKB) is the source of the GWAS data, and the MRC Integrative Epidemiology Unit (IEU) open genome-wide association study (GWAS) research was used to analyze the metabolites in human blood (25, 26).

### 2.3 Outcome of GWAS data

GWAS data on ADHD have been made public by a number of organizations and institutes. In order to choose appropriate GWAS data, the following criteria were set up: The original data structure must comply with the specifications of Mendelian random analysis, specifically the id of SNP, beta, standard error, effect allele, other allele, and *p*-value. It also needs to explicitly state the sample size of the control and case groups. Psychiatric Genomics Consortium (PGC; https://pgc.unc.edu/for-researchers/download-results/), MRC IEU,^1^ Integrative Psychiatric Research (iPSYCH; https://ipsych.dk/en/research/downloads/), and GWAS_Catalog^2^ were the GWAS data that were screened according to the above criteria.

General ADHD outcome data were retrieved from the meta-analysis published within the data repository of the Psychiatric Genomics Consortium^3^ (27), primarily of European descent (38,691 cases and 186,691 controls). Second, the results of the Joanna’s study (28), which is accessible to the public in PGC and iPSYCH, provided the ADHD GWAS for various sexes. In particular, there were 32,102 European participants in the male-only GWAS (14,154 cases and 17,948 controls), and 21,191 people in the female-only GWAS (4,945 cases and 16,246 controls). Third, Rajagopal et al.’s iPSYCH publication provided the summary data of ADHD at various diagnosis times (29). The ICD-10 criteria were used to diagnose ADHD in Europeans. Depending upon the age of the initial diagnosis, it contained three types. The Adulthood ADHD outcome pertains to persons who received their initial diagnosis of ADHD during their adult years (*N* = 6,961). ADHD diagnosis throughout childhood is referred to as childhood (*N* = 14, 878). Being diagnosed with ADHD as a child and then again as an adult is known as persistent ADHD (*N* = 1,473 instances). *N* = 38,303 participants in the control group did not have an ADHD diagnosis. Appendix A and Supplementary Table S1 provide all of the information of the sources of the data used in summary statistics.

### 2.4 The selection of IVs

Instrumental variables (IVs) were used in the MR analysis to find a causal link between exposure and outcome. SNPs, or single nucleotide polymorphisms, were employed in this research as IVs to infer causal relationships between exposure and outcomes. To meet the relevance assumption, SNPs associated with each exposure at genome-wide significance (*p* < 5 × 10^−8^) level were extracted as IVs. Due to the lack of effective IVs and in accordance with previous studies (30–32), we adopted a more lenient significance threshold of 1 × 10^−6^ for antioxidant dietary supplements, such as coffee intake, green tea intake, herbal tea intake, standard tea intake, and average weekly red wine consumption. For the same reason, the *p* value threshold for IVs selection was set at 1 × 10^−5^ for diet-derived circulating antioxidants, such as vitamin C (ascorbate), vitamin E (*α*-tocopherol), vitamin E (*γ*-tocopherol), carotene, vitamin A (retinol), zinc, and selenium. It is noteworthy that the clumping technique was utilized to filter all the SNPs in significant linkage disequilibrium, with a window size of 10 MB for each exposure and a r^2^ threshold of less than 0.001. Besides, it was necessary for the F-statistic (F = Beta^2^/SE^2^; wherein Beta indicates the SNP’s effect size on exposure and SE represents beta’s standard error) to be larger than the typical value of 10, which indicates the strength of the correlation between SNPs and diet-related antioxidant exposures (33). Several SNPs were eliminated as a final step if palindromic structures were found or if they were not included in the outcome summary data.

### 2.5 Statistical analysis

This study used the two-sample Mendelian randomization (MR) methodology to examine the causal connection between diet-related antioxidant exposures and six types of ADHD. Inverse variance weighted (IVW), weighted median, and MR-Egger were among the methods used. In this work, the IVW approach was primarily used, with a meta-analysis summing the Wald estimates of an individual SNP (34). Meanwhile, MR-Egger and weighted median analyses were performed to corroborate the IVW method’s findings. Estimates are produced reliably and effectively by the IVW approach when all genetic variations are taken into account as legitimate. Nonetheless, the weighted median method becomes the best course of action if more than 50% of the genetic variations are deemed invalid (35). The MR-Egger approach is used when all genetic variants are taken to be completely invalid (35). These approaches produce reliable predictions in a wider range of scenarios, despite their lower efficiency.

To examine potential horizontal pleiotropy with genetic tools, a number of sensitivity analyses must be carried out. Through its intercept test, the MR-Egger intercept may identify horizontal pleiotropy and offer a corrected pleiotropy estimate (36). After eliminating outliers, MR pleiotropy residual Sum and outlier analysis (MR-PRESSO) can identify putative peripheral SNPs and offer causal estimates (37). The variability between Wald ratio estimates in genetic instruments was evaluated using Cochrane’s Q test. MR-Steiger filtering is applied one more time to lessen the possible effect of reverse causation. The leave-one-out approach guarantees consistency in the outcomes. We considered the consistency of all MR approaches and utilized IVW as the principal causal effect estimate based on the mentioned analyses. To assess the impact of sample overlap on the significance results, we calculated the sample overlap rate between the relevant exposure and outcome, with an acceptable threshold generally set at up to 10% (38).

In order to address confounders, we reran the MR analysis after eliminating specific IVs that were significantly (*p* < 5 × 10^−8^) related with any potential confounders (blood pressure, blood lipids, body mass index, etc.). This was done after scanning PhenoScanner V2 (39) for pleiotropic SNPs of confounders. A false discovery rate *p* value (FDR), derived using the Benjamini-Hochberg (BH) method, was utilized to account for multiple testing while examining the causal association between dietary antioxidants and ADHD. The FDR, which represents the expected proportion of incorrectly rejected discoveries, effectively identifies true positives while controlling the percentage of type I errors at a given threshold (40). Since this study involved 12 different exposures and 6 different outcomes, the number of tests required was 72. Results were considered significant if the FDR was less than 0.1 (41–44). R software (version 4.2.1) was used for all analyses, and R packages such as MR-PRESSO, MendelianRandomization, and TwoSampleMR were used.

## 3 Results

European groups’ exposure GWAS dates ranged from 2,603 to 428,860 (Supplementary Table S1). As shown in Supplementary Tables S2–S7, there is no indication of weak instrument bias because the *F*-values for each IV are greater than 10.

### 3.1 MR analysis findings

**Figure 2:**
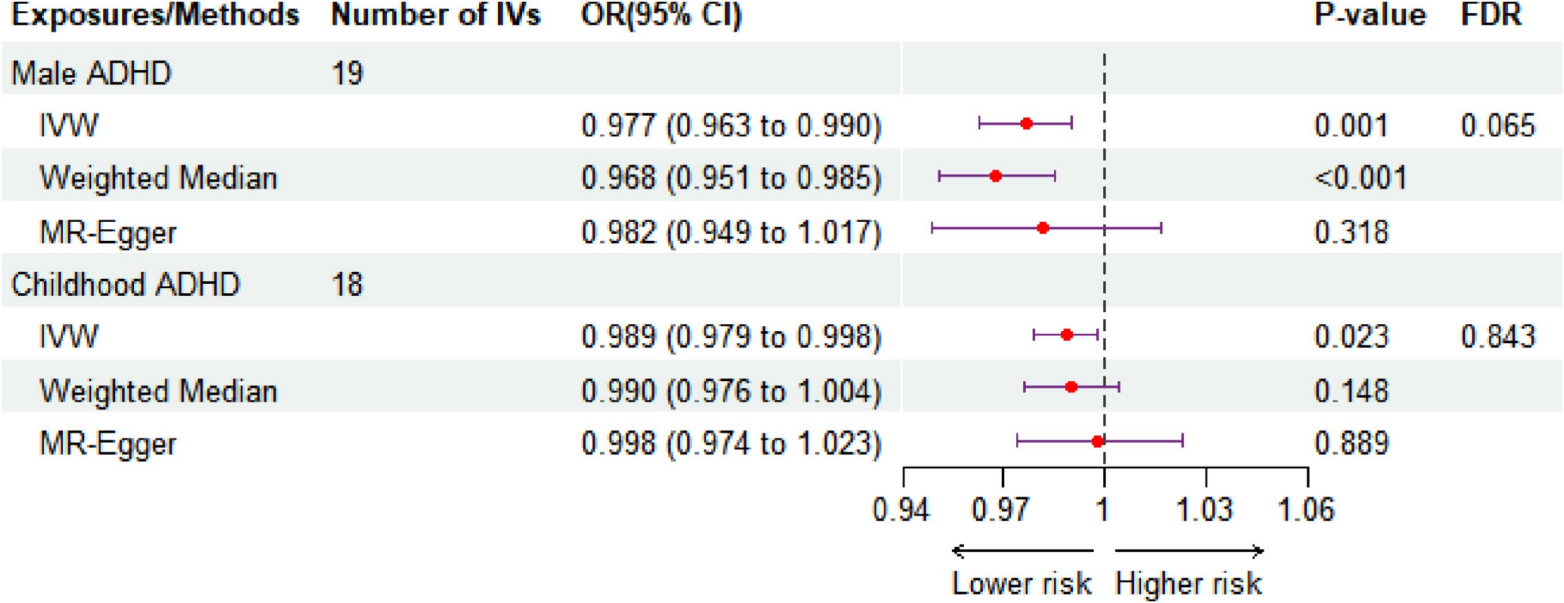
The effect size of green tea intake on the three types of ADHD is revealed by MR analysis. IVW, inverse variance weighted. SNP, single-nucleotide polymorphism; CI, confidence interval; OR, odds ratio; IV, instrument variable; FDR, false discovery rate.

**Figure 3:**
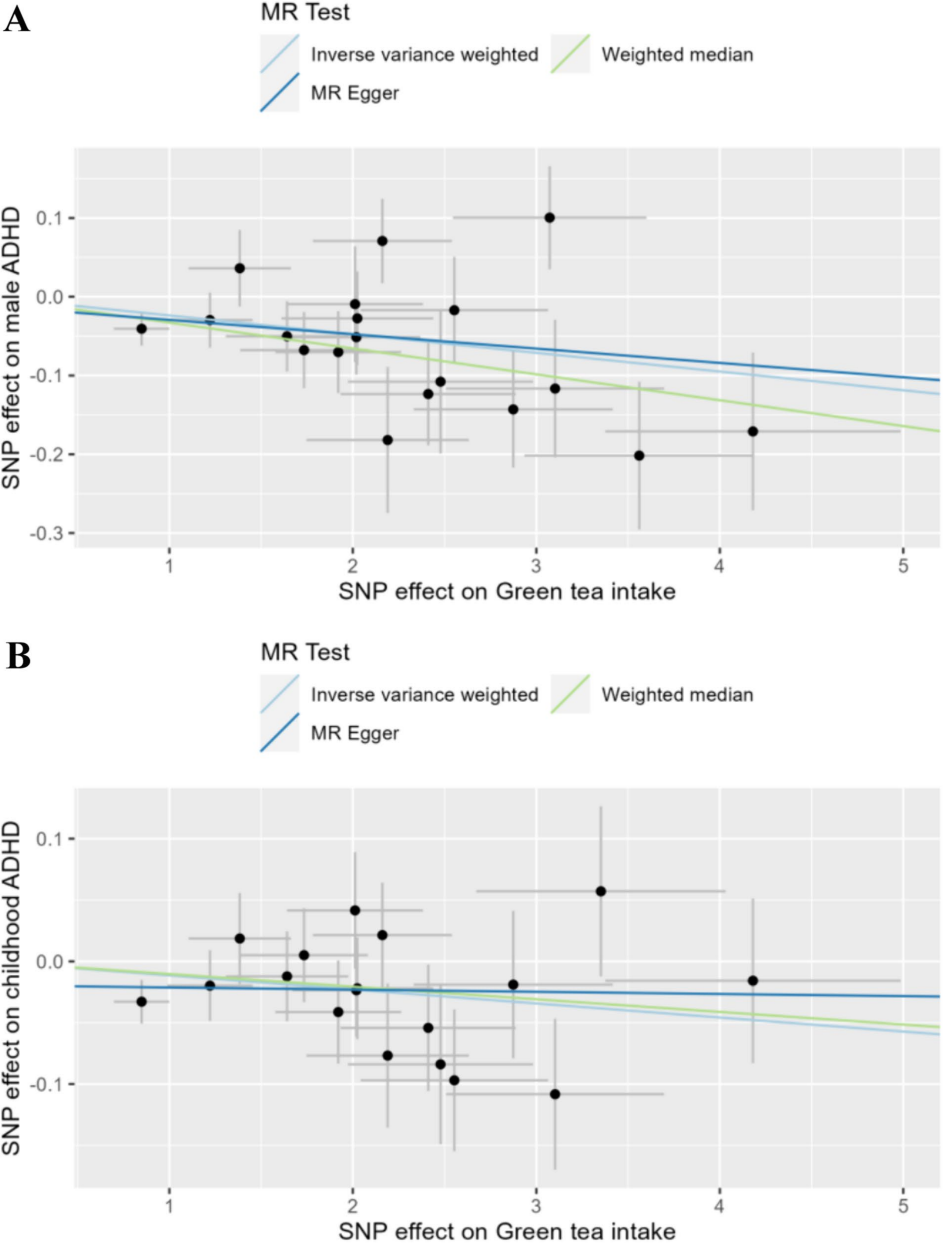
Scatter plot of the association between green tea intake and ADHD. SNP, single-nucleotide polymorphism. **(A)** Green tea vs. male ADHD. **(B)** Green tea vs. childhood ADHD.

The principal analytical approach IVW (OR: 0.977, CI: 0.963–0.990, *p* < 0.001, FDR = 0.065) revealed that intake of green tea significantly reduced the risk of male ADHD after the original *p*-values were corrected using the BH method (Figure 2; Supplementary Table S8). This finding was in line with the weighted median result (OR: 0.968, 95% CI: 0.951–0.985, *p* < 0.001, Figure 2), while MR Egger did not produce statistically significant results (*p* > 0.05, Figure 2). It is worth noting that, without correcting for *p*-values, the IVW method also revealed a possible nominal causal relationship between green tea intake and childhood ADHD. Figure 2 showed that drinking green tea was associated with lower risks of childhood ADHD (OR: 0.989, 95% CI: 0.979–0.998, *p* = 0.023, FDR = 0.843). Figure 3 displays scatter plots showing the association between green tea intake and male ADHD or child ADHD. Regarding other dietary antioxidants, with or without corrected p-values, we were unable to discover any association using the IVW method between them and various kinds of ADHD (Supplementary Table S8).

### 3.2 Sensitivity analyses

Several sensitivity analyses were performed to ensure the validity of the results with significance (negative association between green tea intake and male ADHD). Cochrane’s Q test revealed that the main results were not heterogeneous. No confounding factors affected our results, as shown by the MR-Egger intercept, which did not discover any possible horizontal pleiotropy (*p* > 0.05; Table 1). Furthermore, no outlier SNPs were found in our study by the MR-PRESSO test, suggesting that there is no pleiotropy. Also, we ran a Steiger test, which revealed that the results of the corrections stayed consistent (Table 1). Additional evidence that no estimates were broken comes from the symmetrical funnel plots and the leave-one-out plot, which demonstrate that SNPs had no effect on the result (Supplementary Figures S1, S2). The SNPs used to evaluate the relationship between intake of green tea and male ADHD (19 IVs for male ADHD) were not linked to any possible confounding factors (blood pressure, blood lipids, body mass index, etc.), according to our analysis using the PhenoScanner V2 online tool. According to the literature (26, 28, 38), the sample population overlap between green tea intake and male ADHD is less than 1%.

**Table 1:** Sensitivity analysis of green tea intake and male ADHD.

Exposure	Outcome	No. SNP	Heterogenenity (*p*-value for Q-statistics)	Pleiotropy		MR steiger directionality	
				MR-PRESSO global *p*-value	MR-egger *p*-value	Correct casual direction	Steiger *p*-value
Green tea intake	Male ADHD	19	0.183	0.190	0.735	TRUE	1.09E-16

ADHD, attention-deficit/hyperactivity disorder; MR-PRESSO, Mendelian randomization-pleiotropy residual sum and outlier; and SNP, single-nucleotide polymorphism.

## 4 Discussion

Our study thoroughly evaluated the relationship between 12 dietary antioxidants and six different forms of ADHD. Ultimately, our research revealed potential correlations between green tea intake and male ADHD. The green tea intake was found to significantly lower the risk of male ADHD.

Social and habitual consumption of tea dates back to 3,000 B.C., making it the second most popular beverage after water (45, 46). A member of the Theaceae family, *Camellia sinensis* (L.) originated in China and, by the 17th century, had spread around the world. Differentiating in appearance, taste, chemical content, and flavor, green, oolong, and black teas are all derived from the same plant, *C. sinensis* L. (47) Green tea is made from steam-cooked *Camellia sinensis* leaves without the need for fermentation (48) and is made up of several ingredients, the most significant of which are catechins (30–42% of solid extract weight), of which epigallocatechin gallate (EGCG) is the most prevalent [65%; (49, 50)]. Theanine (4–6%) and caffeine (3–4%) are the next most abundant constituents (48).

It has been shown that EGCG, an active ingredient found naturally in green tea, has neuroprotective properties (51). The anti-inflammatory properties of EGCG are linked to the efficient suppression of cytokine secretory production by microglia, which involves the decrease of proinflammatory cytokines (IL-1β, IL-6, and TNF-α) and the microglia marker Iba-1, hence impeding microglia activation (52–56). TNF-α serves as a primary cytokine involved in inflammatory responses, regulating intraparenchymal signaling and modulating cell growth and survival (51). IL-6 functions as a major mediator of immune and inflammatory responses, primarily activated through STAT3 signaling pathways (57). IL-1β, an agonistically active secretory factor within the IL-1 family, is produced by brain microglia upon TLR activation, leading to decreased neurogenesis and marked reductions in synaptic plasticity (58). A recent observational research indicates that the ADHD group had considerably greater oxidant status and oxidative stress index, while their total antioxidant status was lower (*p* < 0.001) than that of the control group. In a similar vein, the ADHD group had statistically greater levels of IL-1β, IL-6, and TNF-α (4, 59).

L-theanine (N-ethyl-l-glutamine), an analogue of l-glutamine and l-glutamic acid, constitutes up to 50% of all free amino acids in green tea and imparts an umami taste (60). In contrast to the placebo, L-theanine significantly enhanced total cognition composite and sustained attention, with a trend towards reducing inhibitory control, as indicated by stop-signal reaction time (61). Moreover, L-theanine plus EGCG promoted mitochondrial activity and energy metabolism, inhibited pathways leading to inflammation and aggregate formation, markedly raised the percentage of G0/G1 in the cell cycle, downregulated the expression of certain proteins like p-mTOR, Cyclin D1, and Cyclin B1, and upregulated the expression of GAP43, Klotho, p-AMPK, and other proteins. Finally, they had effects on differentiated nerve cells that included repair and regeneration (62). The synergistic mechanism investigation shown that l-theanine might have a nourishing impact on nerves based on the theory that EGCG reduces inflammation and amyloid stress while boosting metabolism.

Sex differences in the prevalence of ADHD are extensively documented in the literature. While at least 11% of children in the United States receive an ADHD diagnosis (63), prevalence ratios between males and females vary considerably, ranging from 3:1 to 16:1 (64, 65). It is notable that the majority of diagnosed youth are male. Male youth with ADHD are more prone to display hyperactive and impulsive behaviors, often leading to expedited referral and diagnosis (66, 67). As a result, a large portion of the research that has already been done on ADHD has primarily or solely involved male youth samples. However, research on how gender might differ in how they react to evidence-based therapies for ADHD has not turned up any conclusive results yet (68, 69). Thus, our results offer novel perspectives on how ADHD is currently managed and how gender variations in its care occur.

The following are our study strengths: first, although RCTs are commonly used in causal studies, their effectiveness is limited by their high cost, complexity, and potential for confounding biases. However, by randomly allocating SNPs at conception, MR analysis efficiently avoids both reverse causality and confounding bias. Secondly, we obtain our auxiliary variables from GWAS databases and recently published studies. With a sample size of more than 420,000, we are able to ascertain the consequence of causality and the genome-wide risk more precisely. Finally, our study addresses the limitations of previous research. While Zhao et al. (70) explored the causal relationship between several dietary-derived circulating antioxidants and six major psychiatric disorders (including anxiety disorders, major depressive disorder, bipolar disorder, schizophrenia, post-traumatic stress disorder, and obsessive-compulsive disorder) using the Mendelian randomization method, their study not only failed to identify any significant associations but also overlooked ADHD, an equally prevalent neuropsychiatric disorder. To fill this gap, we extended their analysis and discovered a potential causal relationship between green tea intake and male ADHD.

## 5 Limitations

There were certain limitations on this study. First, it can be difficult to address epigenetic aspects in MR studies, such as chromatin remodeling, non-coding RNA regulation, and DNA methylation (71). Second, the reliance on summary statistics rather than raw data limited our ability to conduct detailed subgroup analyses, such as examining different ethnicities or age groups within the same gender. Although we had GWAS data for ADHD spanning multiple time periods and genders and observed differences in odds ratios (ORs) between groups, determining the statistical significance of these differences proved challenging. Third, while our study included rigorous sensitivity tests to address and mitigate the effects of horizontal pleiotropy, there may still be unknown biological processes, diseases, or behaviors linked to the selected SNPs. Future research should explore these SNPs further to gain a more comprehensive understanding of their roles in the observed associations. Lastly, even though we have not been able to definitively link any other dietary antioxidants to ADHD just yet, this might be because there aren’t currently enough SNPs in the analysis. When the number of instrumental variables in a given dietary antioxidant rises, significant associations might be discovered, so future research needs to include GWAS databases with larger sample sizes to re-analyze them. Given these limitations, caution is warranted in interpreting our findings. We recommend that future studies, especially randomized controlled trials (RCTs), be undertaken to validate this potentially incidental result and establish a definitive causal relationship.

## 6 Conclusion

In conclusion, our study examined the relationships between 12 dietary antioxidants and 6 types of ADHD. Among the numerous associations tested, we identified a significant causal association between green tea intake and male ADHD, suggesting that increased consumption of green tea may reduce the risk of ADHD in this population. This finding could provide targeted guidance for ADHD interventions. Future studies should focus on the effects of different doses of green tea intake on ADHD in males.

## Data Availability

The original contributions presented in the study are included in the article/Supplementary material, further inquiries can be directed to the corresponding authors.

## Footnotes

^1^https://gwas.mrcieu.ac.uk

^2^https://www.ebi.ac.uk/gwas/summary-statistics

^3^https://pgc.unc.edu/
