# Supplementary material for: Exploring causal associations of antioxidants from supplements and diet with attention deficit/hyperactivity disorder in European populations: a Mendelian randomization analysis
Source: Front Nutr. 2024 Sep 24;11:1415793. doi: 10.3389/fnut.2024.1415793 (PMC11459460; doi:10.3389/fnut.2024.1415793)
Supplement: Supplementary file 2 [file Table_2.docx]

**1. Description of summary statistics data sources**

**1.1** **GWAS of** **coffee intake,** **green tea intake, herbal tea intake,** **standard tea intake, average weekly red wine, carotene, and vit. A (retinol) by** **UK Biobank consortium**

The summary-level data for the phenotypes including coffee intake, green tea intake, herbal tea intake, standard tea intake, average weekly red wine consumption, carotene, and vitamin A (retinol) in European populations originates from the UK Biobank (UKB). This dataset underwent adjustments for age, sex, 10 genetic principal components, and genotyping batch. The UK Biobank, a study primarily focused on the UK, compiled medical and physical information from around 500,000 participants aged between 40 and 69 years, during the period from 2006 to 2010. (1-4)

**Coffee intake:** Data on coffee intake was collected via a questionnaire survey, querying participants about their daily coffee intake, including decaffeinated coffee.

Several checks were implemented to ensure data quality:

Responses less than 0 were rejected.

Responses exceeding 99 were rejected.

Participants reporting more than 10 cups per day were prompted to confirm their response.

If the participant activated the Help button they were shown the message:

Participants are advised to provide an average considering their intake over the past year. In case of uncertainty, they are encouraged to estimate or select "Do not know." Additionally, the coding system incorporates three special values:

"-10" indicates "Less than one cup per day."

"-1" signifies "Do not know."

"-3" denotes "Prefer not to answer." These values assist in accurately capturing and categorizing responses within the dataset.

**Green tea intake:** The inquiry posed was: "How many cups or mugs of green tea, such as Tieguanyin or Longjing, did you consume yesterday?" After excluding low-quality samples, the summary statistics were generated. Green tea consumption in the original questionnaire was classified into 0, 1, 2, 3, 4, 5, and ≥6 cups, with each cup specified as 250 mL, as indicated in the original study (<http://www.nealelab.is/uk-biobank>).

**Herbal tea intake:** Participants were asked: "Yesterday, how many cups or mugs of herbal tea, did you consume?" After excluding low-quality samples, the summary statistics were generated. Green tea consumption in the original questionnaire was classified into 0, 1, 2, 3, 4, 5, and ≥6 cups, with each cup specified as 250 mL, as indicated in the original study (<http://www.nealelab.is/uk-biobank>).

**Standard tea intake:** The query presented was: "Did you consume any tea or infusion yesterday?"

Participants who activated the Help feature were provided with the following message: "Standard tea encompasses all varieties made with black tea leaves, including teabags, loose leaf tea, and decaffeinated types. Peppermint tea belongs in the herbal or fruit infusion section. If you sweetened your tea with honey, consider it as sugar. Artificial sweeteners should not be treated as sugar. If your tea or infusion isn't listed, please specify it under 'Other' located at the bottom of the list."

**Average weekly red wine:** The ACE touchscreen presented the question: "In an average week, how many glasses of red wine do you typically consume? (Note: There are six glasses in an average bottle)"

Several checks were implemented to ensure data quality:

If the reported number of glasses is less than 0, the response is rejected.

If the reported number exceeds 250 glasses, it is considered implausible and rejected.

If the reported number surpasses 100 glasses, the participant is prompted to confirm the accuracy of their response.

If a participant activates the Help button, they were shown the message:

Field 1568 was gathered from participants indicating alcohol consumption more frequently than once or twice a week, as determined by their responses to Field 1558.

Coding 100291 defines 2 special values:

-1 represents "Do not know"

-3 represents "Prefer not to answer"

**Carotene:** Estimated intake, based on food and beverage consumption yesterday, excluding any supplements.

**Vit. A (retinol):** Retinol. Estimated intake, based on food and beverage consumption yesterday, excluding any supplements.

**1.2** **GWAS of Vit. C (ascorbate), Vit. E (α-tocopherol), Vit. E (γ-tocopherol) by Shin SY et al. (5)**

The study utilized genome-wide association studies (GWAS) to pinpoint genetic variants linked with blood metabolite levels. Data sources encompassed the TwinsUK cohort, the KORA cohort, and the Framingham Heart Study.

Vitamin C, or ascorbate, is indispensable for various physiological functions, such as collagen synthesis, wound healing, and immune response. The GWAS on vitamin C levels identified genetic loci associated with this nutrient, notably SNPs in the SLC23A1 and SLC23A2 genes, which encode vitamin C transporters. Vitamin C levels exhibited a heritability of 48%, with common genetic variants elucidating 18% of the variance.

Vitamin E encompasses a group of fat-soluble compounds like α-tocopherol and γ-tocopherol, possessing antioxidant properties vital for cell membrane integrity. The GWAS on vitamin E levels disclosed genetic loci linked with these compounds, including SNPs in the HMGCR and CYP4F2 genes, involved in cholesterol metabolism and fatty acid oxidation, respectively. The heritability of α-tocopherol levels stood at 56%, with common genetic variants clarifying 24% of the variance. The heritability of γ-tocopherol levels was estimated at 54%, with common genetic variants explaining 22% of the variance.

Overall, This study's findings on genetic influences on blood metabolite levels, like vitamins C and E, are crucial for personalized medicine and disease prevention, emphasizing the importance of tailored interventions based on individual genetic profiles.

**1.3** **GWAS of Zinc and Selenium by Evans DM et al. (6)**

In this GWAS, researchers sought to uncover genetic factors influencing blood concentrations of zinc and selenium, essential trace elements vital for human health. Using data from adult cohorts in Australia and the UK, they measured erythrocyte copper, selenium, and zinc levels via inductively coupled plasma mass spectrometry. Genotyping, conducted with Illumina chips, enabled the imputation of over 2.5 million SNPs from HapMap data.

The results revealed significant loci for each element. Copper exhibited two loci on chromosome 1, with rs1175550 and rs2769264 as the most significant SNPs. Selenium displayed a significant locus on chromosome 5, with rs921943 being notable in both cohorts. Zinc showcased three significant loci on chromosomes 8, 15, and X, with rs1532423, rs2120019, and rs4826508 as the most significant SNPs, respectively..

None of the genome-wide-significant SNPs in the study displayed evidence of association with more than one element, even at P < 0.05. However, suggestive SNPs suggested a locus at EPHA6 (EPH receptor A6) potentially influencing both copper and zinc. Two uncommon SNPs at this locus showed suggestive results for one element and significantly low P-values for the other.

Furthermore, gene-based analysis yielded no additional significant loci beyond those identified by allelic association analysis of SNP data. Overall, the study provides valuable insights into genetic variations linked to health effects related to sub-clinical deficiency of essential trace elements. Identifying these loci can aid in preventing deficiency-related health issues and developing personalized nutrition interventions.

**1.4** **GWAS of Attention Deficit/Hyperactivity Disorder (ADHD).**

**General ADHD** is described as a neurodevelopmental disorder marked by hyperactivity and diminished attention, diagnosed by a psychiatrist using the ICD10 diagnosis code F90.0. (7)

**Male ADHD** refers to males diagnosed with ADHD, while **Female ADHD** pertains to females diagnosed with ADHD. These samples originate from Denmark and were collected by the Lundbeck Foundation Initiative for Integrative Psychiatric Research (iPSYCH), along with 11 cohorts pooled by the Psychiatric Genomics Consortium (PGC) from Europe, North America, and China. (8)

**Childhood ADHD** is characterized by cases diagnosed with ADHD under the age of 18 in 2016 or cases over 18 by the end of follow-up in 2016 who did not receive another ADHD diagnosis beyond 18. **Persistent ADHD** refers to cases diagnosed with ADHD as children (before 18) and again as adults (after 18). **Adulthood ADHD** encompasses individuals diagnosed with ADHD as adults, aged 18 and older. These samples were identified in a nationwide population-based case cohort established by iPSYCH. (9)

**2. Reference**

1. Sudlow C, Gallacher J, Allen N, et al. UK biobank: an open access resource for identifying the causes of a wide range of complex diseases of middle and old age. PLoS Med (2015) 12:e1001779.

1. Cornelis MC, van Dam RM. Habitual Coffee and Tea Consumption and Cardiometabolic Biomarkers in the UK Biobank: The Role of Beverage Types and Genetic Variation. J Nutr. 2020;150(10):2772-2788. doi:10.1093/jn/nxaa212
2. Qi J, Zhang K, Zhang P, et al. Habitual coffee and tea consumption and risk of cataract: A prospective cohort study from the UK Biobank. Clin Nutr ESPEN. 2024;62:81-87. doi:10.1016/j.clnesp.2024.05.006
3. Zhang S, Wu S, Xia B, et al. Association of coffee and tea consumption with osteoporosis risk: A prospective study from the UK biobank. Bone. 2024;186:117135. doi:10.1016/j.bone.2024.117135

5. Shin S-Y, Fauman EB, Petersen A-K, et al. An atlas of genetic influences on human blood metabolites. Nat Genet (2014) 46:543–550. doi: 10.1038/ng.2982

6.Evans DM, Zhu G, Dy V, et al. Genome-wide association study identifies loci affecting blood copper, selenium and zinc. Hum Mol Genet (2013) 22:3998–4006. doi: 10.1093/hmg/ddt239

7. Demontis D, Walters RK, Martin J, et al. Discovery of the first genome-wide significant risk loci for attention deficit/hyperactivity disorder. Nat Genet. 2019;51(1):63-75. doi:10.1038/s41588-018-0269-7

8. Martin J, Walters RK, Demontis D, et al. A Genetic Investigation of Sex Bias in the Prevalence of Attention-Deficit/Hyperactivity Disorder. Biol Psychiatry (2018) 15;83(12):1044-1053. doi: 10.1016/j.biopsych.2017.11.026

9. Rajagopal VM, Duan J, Vilar-Ribó L, et al. Differences in the genetic architecture of common and rare variants in childhood, persistent and late-diagnosed attention-deficit hyperactivity disorder. Nat Genet. 2022;54(8):1117-1124. doi:10.1038/s41588-022-01143-7
