# Supplementary material for: Exploring causal associations of antioxidants from supplements and diet with attention deficit/hyperactivity disorder in European populations: a Mendelian randomization analysis
Source: Front Nutr. 2024 Sep 24;11:1415793. doi: 10.3389/fnut.2024.1415793 (PMC11459460; doi:10.3389/fnut.2024.1415793)
Supplement: Supplementary file 3 [file Image_1.pdf]

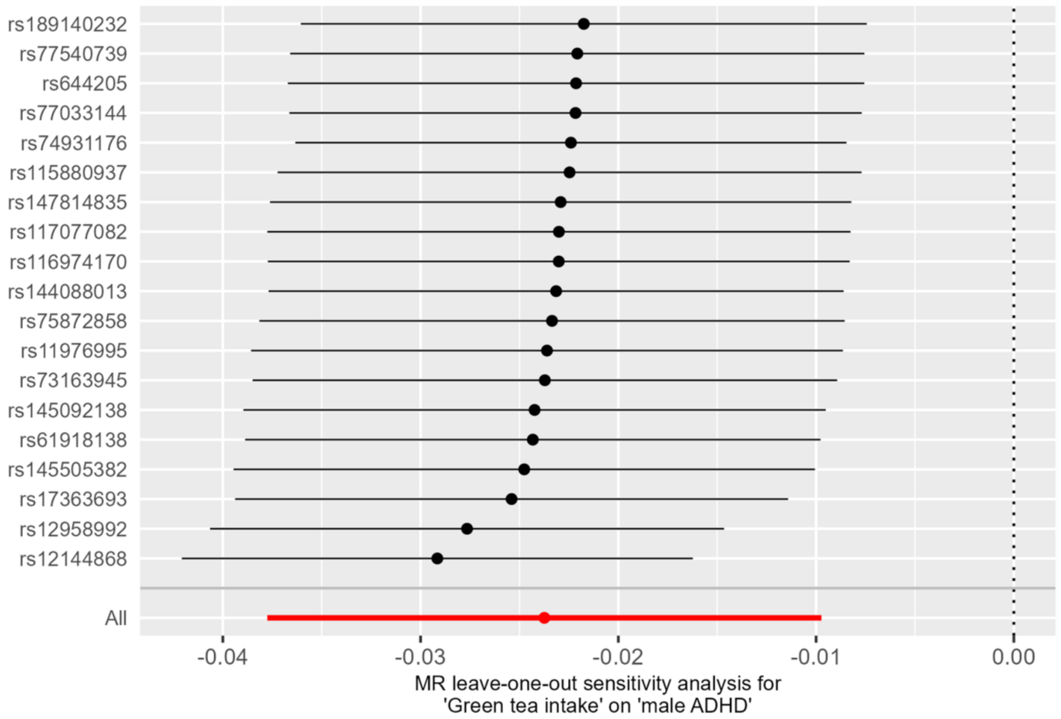

Figure S1. MR leave-one-out sensitivity analysis

Leave-one-out analysis indicates fluctuant associations of green tea intake vs male ADHD.

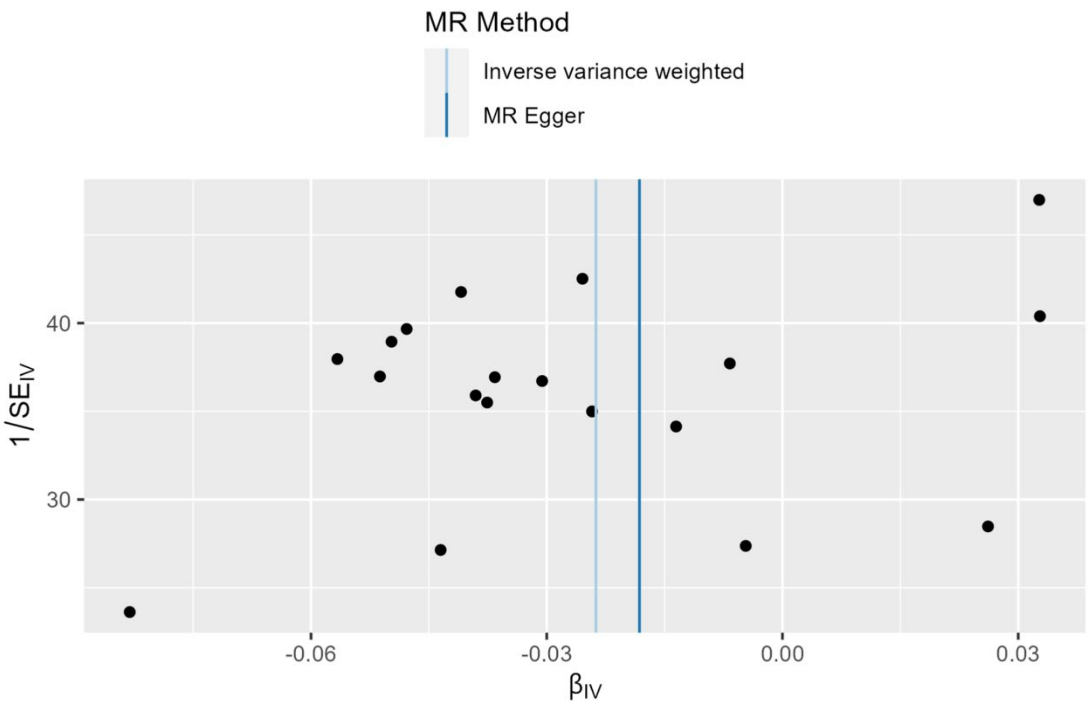

Figure S2. Funnel plots of MR analysis. Green tea intake vs Male ADHD.
